# Magnetization Reversal and Dynamics in Epitaxial Fe/Pt Spintronic Bilayers Stimulated by Interfacial Fe_3_O_4_ Nanoparticles

**DOI:** 10.3390/ma14164354

**Published:** 2021-08-04

**Authors:** Thomas Kehagias, Dimitrios Karfaridis, Camillo Ballani, Laura Mihalceanu, Christoph Hauser, Isaak G. Vasileiadis, George P. Dimitrakopulos, George Vourlias, Evangelos Th. Papaioannou

**Affiliations:** 1 Physics Department, Aristotle University of Thessaloniki, 54124 Thessaloniki, Greece; dkarfari@physics.auth.gr (D.K.); isvasile@physics.auth.gr (I.G.V.); gdim@auth.gr (G.P.D.); gvourlia@auth.gr (G.V.); 2 Institute of Physics, Martin-Luther University Halle-Wittenberg, 06120 Halle, Germany; camillo.ballani@physik.uni-halle.de (C.B.); Chris-Hauser90@web.de (C.H.); evangelos.papaioannou@physik.uni-halle.de (E.T.P.); 3 Department of Physics, Technical University of Kaiserslautern, 67663 Kaiserslautern, Germany; mihalcea@rhrk.uni-kl.de

**Keywords:** spintronic bilayers, magnetite nanoparticles, dislocation pipe diffusion, magnetization reversal, spin pumping, HRTEM, XPS, magnetic measurements, ISHE

## Abstract

We have explored the impact of elevated growth and annealing temperatures on the local interfacial structure of thin Fe(12 nm)/Pt(10 nm) spintronic bilayers, epitaxially grown on MgO (100), and their correlation to magnetization reversal and dynamics. Electron-beam evaporation growth and subsequent annealing at 450 °C causes significant roughening of the MgO/Fe interface with irregular steps and multilevel (100) MgO surface terraces. Consequently, threading dislocations emerging at the step edges propagated in the Fe layer and terminated at the Fe/Pt interface, which appears pitted with pits 1.5–3 nm deep on the Fe side. Most of the pits are filled with the overlying Pt, whereby others by ferrimagnetic Fe_3_O_4_, forming nanoparticles that occupy nearly 9% of the Fe/Pt interfacial area. Fe_3_O_4_ nanoparticles occur at the termination sites of threading dislocations at the Fe/Pt interface, and their population density is equivalent to the density of threading dislocations in the Fe layer. The morphology of the Fe/Fe_3_O_4_/Pt system has a strong impact on the magnetization reversal, enhancing the coercive field and inducing an exchange bias below 200 K. Furthermore, low-temperature spin pumping and inverse spin Hall effect voltage measurements reveal that below their blocking temperature the nanoparticles can influence the spin current transmission and the spin rectification effects.

## 1. Introduction

Progress in nanofabrication techniques for magnetic materials, such as Fe, Ni, Co, and their alloys, over recent decades have enabled the well-controlled growth of nanometer-thick layers and multilayers and led to many discoveries in the field of nanomagnetism and spintronics. Brilliant examples are the giant magnetoresistance (GMR) [1,2] and tunneling magnetoresistance (TMR) effects [3], which have already found their way to everyday life applications. Recently, part of the research on spin transport at and through interfaces focuses on spin pumping effects using microwave fields and thermally induced spin transport, as well as the engineering of multilayer stacks of magnetic and non-magnetic material layers [4,5]. Investigations on the emergence of spin current flow have revealed new physical mechanisms like the spin Hall effect (SHE) [6,7,8]. SHE can lead to spin currents that flow perpendicular to the direction of charge current via spin-dependent scattering processes (intrinsic (or) impurities) in metals with large spin-orbit coupling. The reciprocal effect, known as the inverse spin Hall effect (ISHE) [9,10], refers to the process in which a pure spin current leads to charge accumulation and development of an electromotive force in a direction transverse to the spin current. The effort today is to efficiently enhance the spin current transport using SHE and ISHE, by manipulating interfaces between the layers and subsequently by modifying the static and dynamic interfacial effects associated with the spin-orbit coupling and the intrinsic symmetry breaking at interfaces [11].

In this direction, our work investigates static and spin pumping phenomena in Fe/Pt bilayers with a modified interface. We study the growth of thin Fe/Pt bilayers on MgO (100) substrates aiming to structurally manipulate the Fe/Pt interface by the growth and annealing temperatures. Fe/Pt multilayers and their alloys are widely investigated for interface-induced effects like magnetic proximity effect (MPE) [12,13] and interface-induced anisotropies. When Fe and Pt layers are a few monolayers thick, they can exhibit perpendicular anisotropy [14], while alloyed structures like the ordered tetragonal Fe-Pt alloy (L10) possess the highest magnetocrystalline anisotropy [15]. The Fe/Pt system has recently attracted further attention in magnetic dynamics studies, since the large spin-orbit coupling in Pt gives rise to spin-dependent Hall effects, when in contact with a magnetic layer. Spin pumping measurements in Fe/Pt bilayers revealed an efficient spin transport through epitaxial interfaces [16,17,18], the role of two-magnon scattering in the quantification of the spin mixing conductance [19], while the Fe/Pt bilayers are perfect candidates for THz-sources based on the spin to charge conversion via the ISHE [20,21].

Here, we focus on modifying the Fe/Pt interface by growing and annealing the bilayer system at the elevated temperature of 450 °C. Besides the epitaxial growth of the entire configuration, High Resolution Transmission Electron Microscopy (HRTEM) imaging along with Fast Fourier Transformation (FFT) diffractograms, HRTEM image simulations, and Geometric Phase Analysis (GPA) [22], as well as X-ray Photoelectron Spectroscopy (XPS) analysis, revealed the formation of Fe_3_O_4_ nanoparticles at the Fe/Pt interface. These temperature induced nanoparticles, although they cover only about 9% of the interfacial area, are able to affect the magnetic order and stimulate interfacial effects, such as exchange bias, and modify the spin current transmission of the Fe/Pt interface, thus paving the way for manipulating interfaces in magnetic multilayers.

## 2. Experimental Methods

Samples were grown on MgO (100) substrates by electron-beam evaporation under a base pressure of 4 × 10^−10^ Torr. The substrate’s surface was initially cleaned in organic solvents, followed by annealing at 650 ^ο^C for 1 h. The bilayers were then deposited with a rate of 0.3 nm/s at a substrate temperature of 450 °C, followed by 30 min annealing at the corresponding growth temperature, resulting in a Fe(12 nm)/Pt(10 nm) bilayer configuration.

Nanostructural properties of the interfacial structures were investigated by HRTEM, in a Jeol 2011 UHR electron microscope, with a 0.19-nm point resolution and C_s_ = 0.5 mm, operated at 200 kV. Specimens for cross-sectional HRTEM observations were prepared by the standard sandwich technique, followed by automated tripod polishing and final thinning to electron transparency by mild Argon ion (Ar+) milling in the Gatan PIPS.

XPS analysis of the samples was performed on a Kratos Analytical AXIS UltraDLD system, with aluminum monochromatic X-Ray source (E_photon_ = 1486.7 eV), under a chamber pressure of 10^−8^ Torr. The etching process was performed by an Ar+ source applying nominal etching rate of 100.12 Å/min, under 4 kV of accelerating voltage. Wide-scan spectra (full range) were recorded with passing energy of 160 eV, while high-resolution (HR) regions with passing energy of 20 eV during a three-sweep scan.

The magnetic anisotropy and static magnetic properties were probed by magneto-optical Kerr effect microscopy in the longitudinal alignment (L-MOKE). The low-temperature magnetic characterization was done with a Quantum Design SQUID VSM magnetometer.

For the dynamic magnetic study, we performed ISHE measurements. Ferromagnetic resonance (FMR) was excited by microwave magnetic field *h*_rf_ induced by a coplanar waveguide. The Pt layer was facing the antenna using a thin insulating layer between. A tunable static external magnetic field *H* was applied in the x–y plane. Using the lock-in amplifier, we recorded the generated *V*_DC_ voltage in the y-direction.

A more detailed section of the experimental methods is given in the Supplementary Material.

## 3. Results and Discussion

### 3.1. Morphology and Nanostructure

**Figure 1:**
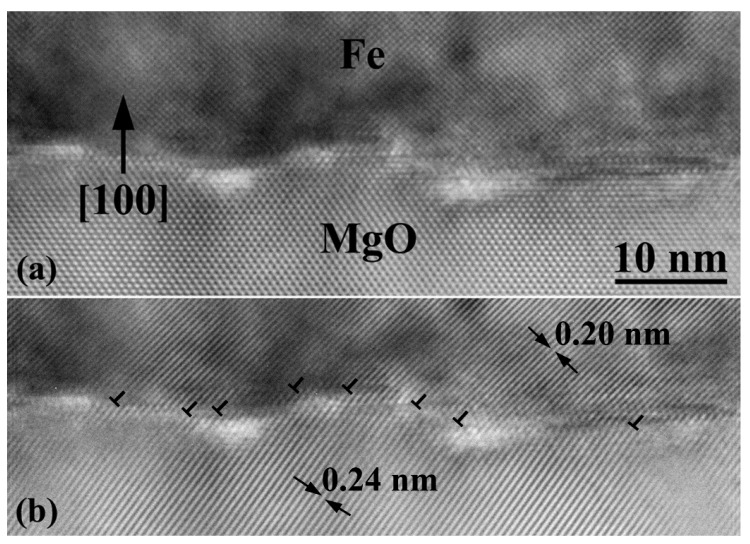
(**a**) HRTEM image of the MgO/Fe interface viewed along the [011]MgO/[001]Fe zone axis. A rough interface integrating steps and (100) terraces is observed. (**b**) A Bragg filtered image of (**a**), showing the coincidence of the MgO {111} (*d* = 0.2427 nm) and Fe {110} (*d* = 0.2027 nm) crystal planes at the interface, where misfit dislocations (here, viewed inclined) are inserted to accommodate the lattice mismatch.

HRTEM imaging of the Fe(12 nm)/Pt(10 nm) bilayer configuration grown on MgO at 450 °C revealed significant findings at the interfacial structures. The MgO/Fe interface is not planar but comprises irregular steps of two to five monolayers height, forming multilevel (100) terraces, as shown in Figure 1a. The Fe layer is epitaxially grown on MgO by a 45° in-plane rotation (Bain orientation), and the resulting lattice mismatch is effectively accommodated by misfit dislocations [23]. When viewed along the [011]MgO/[001]Fe projection direction as in Figure 1, the extra half-planes of misfit dislocations are inclined along the <110> Fe directions, as shown in the Bragg filtered image of Figure 1b. Misfit dislocations bend at the step edges of the MgO/Fe terraces, becoming threading dislocations that propagate along the Fe layer and terminate at the Fe/Pt interface. The Pt layer is also epitaxially grown on MgO/Fe, [011](100)MgO/[001](100)Fe/[011](100)Pt, occasionally deviating from the exact epitaxial orientation relationship by a 1°–3° out-of-plane tilt off the growth axis and toward the [0-11] direction. No threading dislocations were observed in the Pt layer.

Previous studies have shown that annealing of the MgO substrate at temperatures ranging from 500 to 650 °C results in a smooth and atomically flat surface [23,24,25,26,27]. Hence, the occurrence of uneven terraces at the MgO/Fe is attributed to a local disordering of the MgO surface, due to the elevated growth temperature and the subsequent annealing of the Fe layer that favors Mg outdiffusion [24,25,26,27]. There is, however, a fair possibility that part of this high roughness occurs due to decomposition of magnesium hydroxide (brucite), that might exist on the MgO surface, during the pre-annealing of the MgO surface [25]. Furthermore, the presence of residual brucite crystals on the MgO surface, prior to the Fe epitaxy, cannot be excluded. While no such crystals were detected by cross-sectional TEM imaging at the MgO/Fe interface (Figure 1), it can be argued that they decomposed during the high temperature epitaxy of Fe by direct reaction with the overlying Fe species.

On the other hand, since no iron oxide layer was detected at the MgO/Fe interface (Figure 1), outgassing of surface oxygen is anticipated. However, magnesium cannot find its way across the bilayer, since Fe acts as a barrier to Mg diffusion [26,28], and therefore it most likely migrates at the MgO/Fe interface. Conversely, oxygen ions may find diffusion paths through the Fe layer along open-core threading dislocations emanating at the step edges and thus, reach the Fe/Pt interface [29,30,31]. Dislocation pipe diffusion is a well-established mechanism observed in a variety of metals and alloys, which is triggered during thermal activation of the diffusion process in crystalline solids, in the presence of line defects, and appears to accelerate migration of impurities relative to bulk diffusion [32,33,34].

The existence of oxygen across the Fe layer and at the Fe/Pt interface was confirmed by XPS depth-profiling, performed using the Ar+ ion gun. The analysis revealed the existence of nearly 4% oxygen (at.%) in the interfacial Fe/Pt area, primarily on the Fe side and rapidly quenching toward the Pt side.

**Figure 2:**
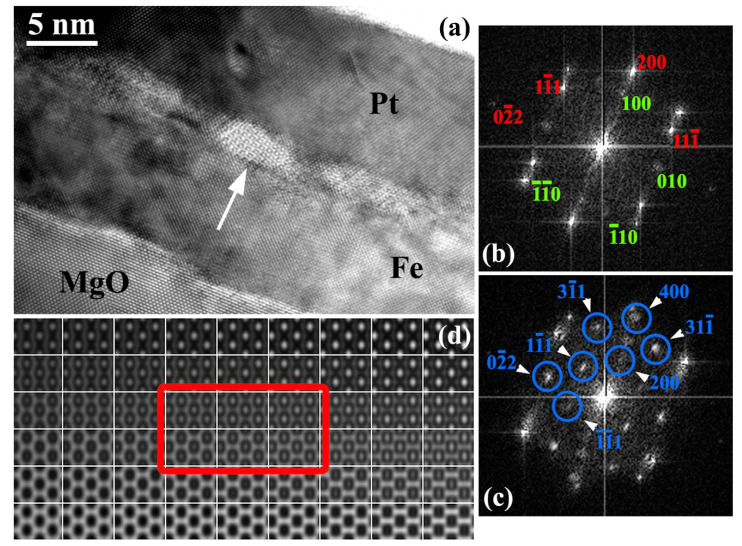
(**a**) HRTEM image of the Fe/Pt interface, along the [011]MgO/[001]Fe/[011]Pt projection direction, comprising three pits. Two of them are filled with Pt, while the third one (white arrow) is filled with ferrimagnetic Fe_3_O_4_. (**b**) Common FFT diffractogram of MgO (red indices) and Fe (green indices). (**c**) Common FFT diffractogram of Fe/Pt along with the central pit. The spatial frequencies denoted by blue circles and their symmetric relative to the central spot belong to the [011] zone axis of Fe_3_O_4_ (magnetite). (**d**) Through-focus-thickness HRTEM image simulation map of magnetite for −46 to 0 nm defocus values (x-axis) and sample thickness from 2.4 to 16.6 nm (y-axis). Image simulations that resemble the experimental image are included in the red rectangle.

Cross-sectional HRTEM imaging showed that the Fe/Pt interface appears rough and heavily pitted on the Fe side, comprising pits with a length of 5–15 nm along the [010]Fe/[0–11]Pt direction and 1.5–3 nm deep. In Figure 2a, a HRTEM image of the Fe/Pt interface is depicted, illustrating three pits, viewed along the [011]MgO/[001]Fe/[011]Pt zone axis. The atomic structure of the two pits on both sides of the central one seems to be a projection of superimposed crystal structures. By digitally subtracting the Fe spatial frequencies, the pits exhibited the Pt crystal structure, suggesting that the atomic structure observed is a consequence of Fe and Pt overlapping projected structures, in front and/or behind the pits, depending on the randomness of the cross-sectional cut of the specimen. Furthermore, GPA phase and strain analysis showed a strain-free Fe/Pt bilayer configuration, meaning that the lattice mismatch has been fully accommodated by misfit dislocations. A detailed example of this experimental process is given in the Supplementary Material (Figure S1).

However, the structure of the central pit (white arrow) exhibits an entirely different symmetry that cannot be associated with either Fe or Pt. We performed an analysis of spatial frequencies of lattice planes using FFT diffractograms, which are shown in Figure 2b,c and explained in the following: Figure 2b is the expected common diffractogram of MgO/Fe along the specific projection direction, while Figure 2c is the diffractogram of the Fe/Pt interface including the central pit’s spatial frequencies shown in blue circles. According to our calculations, involving the evaluation of interplanar spacings compared to the *d*-spacing values of the related lattices, these should belong to the [011] zone axis of stoichiometric Fe_3_O_4_ (magnetite) with an F*d-3m* structure. The 0–22 reflection of Fe_3_O_4_ (d = 0.2969 nm) practically coincides with the 0–10 double diffracted reflection of Fe (d = 0.2866 nm), whereas the forbidden 200 reflection appears due to double diffraction. Remarkably, the Fe_3_O_4_ nanoparticles’ orientation is consistent with those of MgO and Pt, namely [011](100)MgO/[001](100)Fe/[011](100)Fe_3_O_4_/[011](100)Pt. Moreover, another intriguing cross-sectional TEM observation is that magnetite nanoparticles occur at the termination sites of threading dislocations of the Fe layer.

The formation of Fe_3_O_4_ nanoparticles could be reasonably explained either by the direct reaction of residual brucite crystals of the MgO surface with Fe during its high temperature epitaxy, or by oxygen nanopipe diffusion from the MgO surface and its thermal reaction with the Fe layer at the Fe/Pt interface.

The presence of other Fe oxides, such as *γ*-Fe_2_O_3_ (maghemite), is excluded, given that the reduction of symmetry from the cubic inverse spinel lattice of magnetite to the primitive of maghemite, despite the almost identical lattice constants, would produce additional double diffracted reflections that are not observed in our TEM experiment [35]. To consolidate the occurrence of Fe_3_O_4_, HRTEM image simulations were performed using the JEMS software [36]. In Figure 2d, a through-focus-thickness HRTEM image simulation map is displayed. The experimental image of the atomic structure of the Fe_3_O_4_ nanoparticle complies well with a specimen thickness of 10.7–11.9 nm and a defocus value between −20 to −24 nm, shown by the red rectangle in Figure 2d. These values really make sense, since the thickness is compatible with our TEM foil’s thickness, while the defocus values are close to Scherzer defocus, where all structures exhibit the optimal contrast.

**Figure 3:**
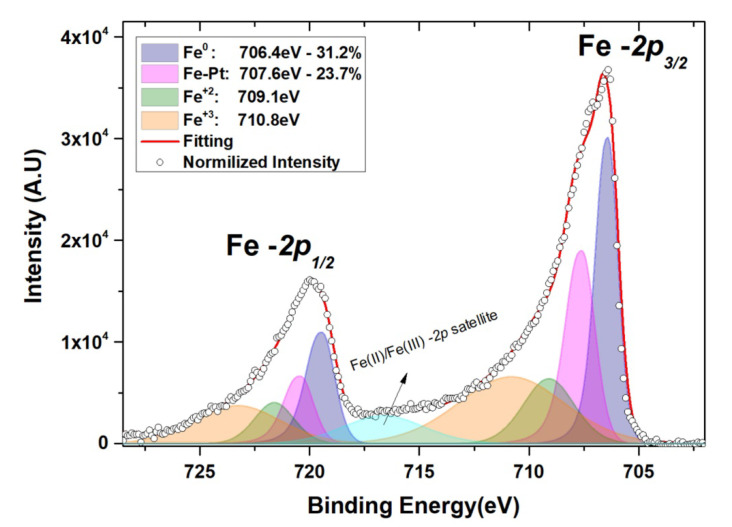
HR peaks of Fe -*2p* orbitals at 12 nm of depth. The deconvolution reveals a four-contribution peak for each of the *2p*_3/2_ and *2p*_1/2_ photoelectron signals, providing the oxidation states of Fe, along with the bimetallic interactions from a relatively significant rough interface.

Furthermore, in the XPS, the deconvolution analysis of the HR peaks from the *2p* orbitals of Fe revealed bonds of the metal with oxygen at the upper Fe surface (Figure 3). Three different oxidation states from Fe were detected. The metallic Fe^0^ at 706.4 eV, the bivalent Fe^+2^ at 709.1 eV, and the trivalent Fe^+3^ at 710.8 eV for the binding energies of the Fe -*2p*_3/2_ orbitals [24,37,38]. The Fe^+2^/Fe^+3^ ratio was calculated by the relative sensitivity factors and the corresponding peak-area, at ≃ 0.476, which along with the presence of the two oxidation states, pinpoints the formation of a near-stoichiometric Fe_3_O_4_ structure (0.5 for stoichiometric [39,40]). The formation of a second contribution, centered at 707.7 eV, presages the bimetallic spin interactions at the Fe/Pt interface [23,41], providing hints of a noteworthy rough interface, as observed in the HRTEM images. An average quantification of Fe -*2p* peaks was performed at the oxidized interface area, by simulations adjusting each ionic state peak area to its concentration in the metallic structure. For depths from between 8.5 and 11 nm (four consecutively intermediate etching steps), the average quantitative calculations used to correct the Fe^+2^/Fe^+3^ ratio, resulted in a ratio of 0.485, which is even closer to the stoichiometric Fe_3_O_4_ configuration.

**Figure 4:**
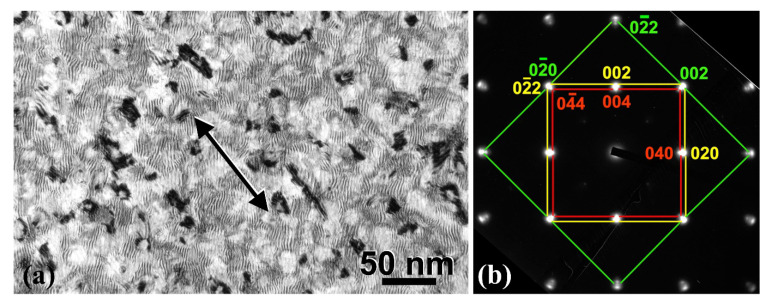
(**a**) Plan-view TEM micrograph showing the distribution of Fe_3_O_4_ nanoparticles at the Fe/Pt interface, along the [100] Fe-Fe_3_O_4_-Pt projection direction. Parallel moiré fringes emerge as a result of overlapping of the Fe and Pt slightly mismatched lattices. (**b**) The corresponding common SAED pattern, showing the epitaxial relation of the three involved lattices (Fe: green, Pt: yellow, Fe_3_O_4_: red) along the growth axis. The lateral side of nanoparticles is oriented roughly along the <0–10>Fe/<0–11>Pt direction, shown by the arrow in (**a**), whereby lateral dimensions greater than 10–15 nm are due to bunched nanoparticles.

TEM plan-view measurements were carried out to estimate the density of Fe_3_O_4_ nanoparticles and the degree of Fe surface area coverage. Plan-view specimens were perforated by ion-milling from the substrate side. In addition, a few seconds of ion-milling was also conducted from the Pt surface side to reveal the nanoparticles. In Figure 4a, a plan-view TEM micrograph of the bilayer is shown, depicting the distribution of the dark contrasted Fe_3_O_4_ nanoparticles at the Fe/Pt interface. The corresponding selected area electron diffraction (SAED) pattern illustrates the epitaxial relation along the [100] growth axis of Fe, Pt, and Fe_3_O_4_ lattices, denoted by green, yellow, and red lines, respectively (Figure 4b). The lateral side of nanoparticles is aligned along the <0–10>Fe/<0–11>Pt direction, shown by the arrow in Figure 4a, as also observed in cross-sectional HRTEM. Translational moiré fringes arise due to the superposition of Fe and Pt crystal lattices, whereby the arc-shaped reflections of Pt imply small misorientations from the exact epitaxial relationship. A population density of 8.9 × 10^10^ Fe_3_O_4_ nanoparticles per cm^2^ was measured, corresponding to ≃ 9% coverage of the Fe/Pt interfacial area. It is worth mentioning that the density of the nanoparticles is equivalent to the average density of threading dislocations in the Fe metallic film. This, in conjunction with the fact that the nanoparticles are localized at the termination sites of threading dislocations of the Fe layer, provides evidence that their origin points towards the oxygen dislocation nanopipe diffusion model.

### 3.2. Magnetic Characterization

**Figure 5:**
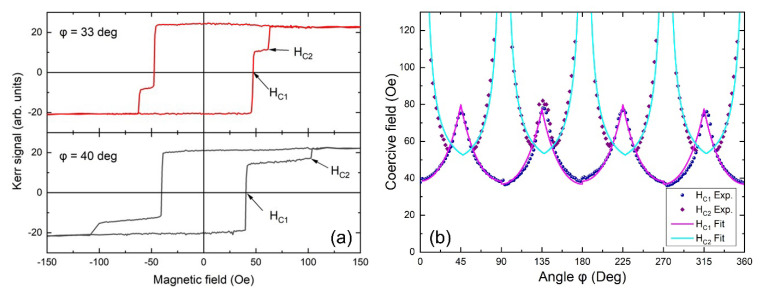
L-MOKE characterization: (**a**) Hysteresis loops with two steps at switching fields *H*_C1_ and *H*_C2_ indicative for two *φ* angles, where *φ* is the angle between the applied magnetic field and the easy magnetization [100] axis of Fe. (**b**) Coercive fields *H*_C1_ and *H*_C2_ plotted against the angle *φ* (data points) and fittings (lines) according to Equation (1). The excellent quality of the fittings indicates the validity of the model of Equation (1).

L-MOKE Kerr effect measurements, at room temperature (RT), show a strong impact on the magnetization reversal of Fe, due to the capping Pt layer and the presence of Fe_3_O_4_ nanoparticles at the interface. The observed hysteresis loops are shown in Figure 5a. The loops are recorded by in-plane rotation of the external magnetic field from *φ* = 0° to 360°, where *φ* is the angle between the applied magnetic field and the [100] easy magnetization axis of Fe. In Figure 5a, one can see two-step hysteresis loops at different in-plane angles, as indicated, where we define the switching field values *H*_C1_ and *H*_C2_ (see Figure 5a). The appearance of two *H*_C1,2_ fields is attributed to the presence of an additional uniaxial magnetic anisotropy overlaying on the cubic fourfold symmetry of the Fe layer as modified by the Pt layer [23]. Under the assumption of the presence of single-domain states with minimum domain wall nucleation energy at *φ* = 0°, 90°, 180°, and 270°, the coercive fields *H*_C1_ and *H*_C2_ can be described by [23,42]:

(1) $$H_{C1,2}=\frac{\frac{\varepsilon_{90{}^{\circ}}}{M}\pm \frac{K_{u}}{M}}{\pm \cos(\varphi )\pm \sin(\varphi )}+H_{off}$$

where *ε*_90°_ is the 90° domain wall nucleation energy and K_u_ is the uniaxial anisotropy constant. The excellent quality of the fitting based on Equation (1) is apparent in Figure 5b, where the coercive fields *H*_C1_ and *H*_C2_ are plotted against the angle *φ*. According to Equation (1), the fitting is possible only if an offset value *H_off_* is added. The last indicates the occurrence of another factor that affects the hysteresis and cannot be associated with the magnetization reversal based only on the magnetic anisotropy of the Fe layer. We attribute the appearance of the term *H_off_* to the presence of Fe_3_O_4_ nanoparticles and Pt-filled pits at the Fe/Pt interface. The nanoparticles, although being a mere 9% of the whole interfacial area, can act as pinning centers for the magnetization reversal and the 90^ο^ domain wall rotation, setting up a lower field limit for the start of the magnetization reversal.

**Figure 6:**
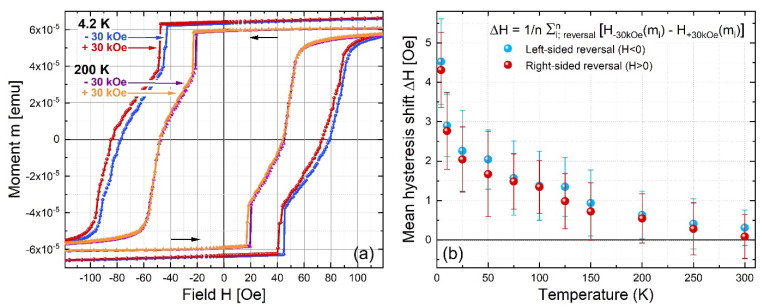
(**a**) Magnetization reversal loops (maximum fields: +2 kOe **→** −2 kOe **→** +2 kOe) measured after cool-down from 400 to 200 K, and from 400 to 4.2 K, respectively. For each temperature, the cool-down is done twice, with initially positive (+30 kOe) and negative (−30 kOe) in-plane fields, respectively. Below 200 K a distinct shift and a small change of the reversal curve shape between positive and negative field-cooled loops is observed. (**b**) Temperature dependent mean hysteresis shift between −30 kOe and +30 kOe field-cooled magnetization reversal curves. For a reliable quantification of the shift with respect to the change in the reversal curve’s shape (Figure 6a), field shifts for multiple fixed moments (within the range of magnetization reversal) are averaged instead of using the difference of the coercive fields (at m = 0) only. This calculation is separately done for both magnetization reversals, *H* < 0 and *H* > 0, respectively, and shows the same sign and a comparable size. The shift increases towards lower temperature, indicating the presence of a growing exchange bias at the Fe/Fe_3_O_4_/Pt interfaces.

To further investigate the magnetic properties and the role of the interfacial nanoparticles, we have performed SQUID measurements from RT down to 4.2 K. In Figure 6a, examples of hysteresis curves obtained from the SQUID magnetometer are shown for two temperatures: 4 and 200 K. The measurement protocol includes initially cooling down in field (3 T), setting the temperature value and then magnetic hysteresis loop run from 2000 to −2000 Oe and back to 2000 Oe. This way, we exclude any experimental factor that can influence the magnetization reversal. A horizontal shift of the hysteresis curves is observed below 200 K. This shift is calculated, using the equation shown in Figure 6b, for the different temperatures, and obtains its maximum value of 4.5 Oe (mean value) at 4.2 K. Hence, the presence of an exchange bias at the Fe/Fe_3_O_4_ nanoparticles/Pt interface, where the exchange bias refers to the zero-field axis shift of the hysteresis loop, is anticipated.

In thin magnetite layers, a strong anisotropy has been observed [43,44]. Isolated Fe_3_O_4_ nanoparticles are widely studied for magnetic hyperthermia and are superparamagnetic below 12–15 nm at RT. Given the size of around 5–15 nm, our Fe_3_O_4_ nanoparticles are classified as superparamagnetic at RT, if isolated. However, here the nanoparticles are embedded in the Fe/Pt interface, and they are magnetically coupled to Fe as the hysteresis loops reveal. The appearance of a shift on the hysteresis reveals that the nanoparticles become magnetically active as individual particles having their blocking temperature below 200 K (Figure 6b). Below this temperature, we observe an increasing shift of the loop hinting at a nanoparticle-induced exchange bias, due to interfacial magnetic coupling between Fe and the nanoparticles. At temperatures still above the blocking temperature, the interfacial nanoparticles’ spins are ferromagnetically coupled to the FM spins. Below the blocking temperature, the nanoparticles’ spins are activated, and they do not follow the applied magnetic field during the magnetization reversal, resulting in a horizontal shift in a magnetization loop.

**Figure 7:**
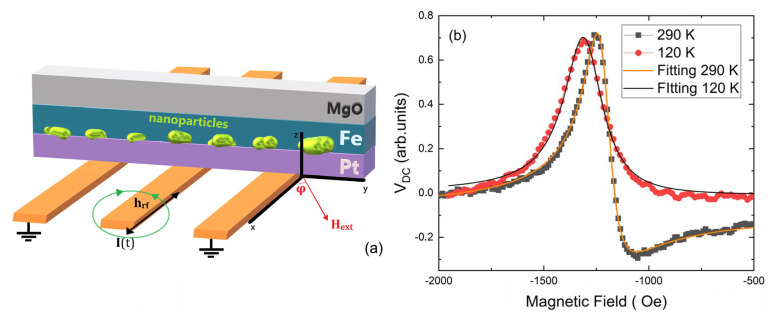
(**a**) The geometrical arrangement of the ISHE measurements. (**b**) ISHE-voltage against the external magnetic field *H* compared for two temperatures: 290 and 120 K. The measurements are shown for *φ* = 0. The excitation frequency was 11 GHz. The lines are fitting curves according to Equation (2). The low-temperature measurement reveals a drastic change in the shape of the signal having a pronounced symmetric part. The latter hints the suppression of spin rectification effects.

Next, we examine the magnetization dynamics properties of the samples by performing spin pumping experiments. In spin pumping experiments, the magnetization of a ferromagnetic layer (FM) in contact with a non-magnetic one (NM) is excited by a microwave field. In such a way, a spin current is generated in the FM layer (Fe) and injected into the NM layer (Pt), and its magnitude is maximized when the FMR condition is fulfilled. The spin current can be detected using the ISHE for conversion into a charge current in appropriate materials. The investigation of the ISHE was performed in a cryostat able to reach temperatures down to 120 K. The goal was to quantify the spin transmission through the specific interface, where nanoparticles are present. The ability of the cryostat to reach temperatures below 200 K that is previously estimated as the limit for the magnetic activation of the particles, enables us to study the influence of the spin current transmission having the nanoparticles at different magnetic states. The detection geometry is shown in Figure 7a, and it includes a static external field, *H*, that rotates in-plane, while the angle *φ* is defined as the angle between the length of the waveguide and *H*. Furthermore, an in-plane dynamic magnetic field *h*_rf_ is generated by the waveguide transverse to the waveguide due to the microwave current *I*(t). The contacts for the voltage detection are transverse to the direction of the waveguide at *φ* = 90°.

In Figure 7b, the detected DC Voltage (*V*_DC_) is presented as a function of the applied magnetic field *H* at two different temperatures: 290 K (defined as RT) and 120 K. The measurements are shown for *φ* = 0°. The measured signal contains voltage contributions from the ISHE and DC voltages induced around the ferromagnetic resonance field (HFMR) by the so-called rectification effects like the anisotropic magnetoresistance (AMR) and the anomalous Hall effect (AHE) [18]. In order to analyze the different *V*_DC_ contributions on the signal, the experimental data are fitted by an expression composed of symmetric and antisymmetric Lorentzian functions [45,46]:

(2) $$V_{DC}(H)=V_{0}+V_{Sym}\frac{{\left(\Delta H\right)}^{2}}{{\left(H-H_{FRM}\right)}^{2}+(\Delta H)}+V_{Asym}\frac{-\Delta H(H-H_{FRM})}{{\left(H-H_{FRM}\right)}^{2}+{\left(\Delta H\right)}^{2}}$$

where *V*_0_ is a constant offset and Δ*H* is the linewidth of the Lorentzian fit curve.

Symmetric contributions to the DC voltage (*V*_Sym_) are expected from the ISHE, the AMR and the AHE [8,20]. Rectification effects emanating from AMR and AHE, contribute only in an antisymmetric manner, *V*_Asym_. In our measurement geometry (Figure 7a), we have the electrical contacts for measuring the DC voltage, the in-plane dynamic field (*h*_rf_), as well as an induced electrical current due to the geometry of the coplanar line, along the y-direction. According to this arrangement, we expected that when the static external magnetic field is oriented along the x-direction, where *φ* = 0°, the contributions from the AMR and AHE rectification effects will be minimized and the signal should be dominated by the ISHE symmetric voltage *V*_Sym_-ISHE, which obtains its maximum value [45,46,47]. However, the *V*_DC_ curve, at RT, acquires a rather asymmetric form implying that the rectification effects are still present. By calculating the ratio of *V*_Sym_/*V*_Asym_, we can get a first estimation of the *V*_DC_ composition. At RT, the *V*_Sym_/*V*_Asym_ ratio is as low as 1.6 ± 0.1. Potential reasons for this behavior can be, for example, possible misorientation of the sample with respect to the waveguide, some additional rectification effects induced by the small out-of-plane microwave magnetic field *h_rf_* along the z-direction, or it may suggest a role of the nanoparticles, since they deteriorate the pure Fe/Pt interface. Interestingly, the measurement at 120 K, reveals a striking difference in the shape of the *V*_DC_ compared to RT. The antisymmetric part of *V*_DC_ seems to almost vanish with the *V*_Sym_/*V*_Asym_ ratio being 14.1 ± 0.1. The drastic change of the line shape and the much larger symmetric contributions at 120 K certainly pinpoint the active role of the nanoparticles in spin transmission through the Fe/Pt interface and minimize other possible geometrical reasons for the quenching of spin rectification effects at lower temperatures, since the geometry remains the same.

Besides that, we have already seen that the nanoparticles below 200 K become magnetically active and induce a small exchange bias. Furthermore, in addition to its magnetic properties, it is known that bulk Fe_3_O_4_ exhibits a conductivity transition from high values at RT to two orders of magnitude lower values at T_V_ ≃ 120 K, the so-called Verwey transition [48]. The metal–insulator transition can also be important for controlling the spin current transmission through the Fe/Pt interface. In short, our results show that Fe_3_O_4_ nanoparticles have the potential, with their fascinating magnetic and electric properties, to interfere with the spin dynamics of the ferromagnetic layer and to tune the spin current transmission in FM/NM interfaces.

## 4. Conclusions

Our findings present a novel route to manipulate interfaces in magnetic multilayers. By using high enough growth and annealing temperatures, the growth of Pt on Fe can be epitaxial. Moreover, under the same conditions, the formation of 8.9 × 10^10^ Fe_3_O_4_ nanoparticles per cm^2^, corresponding to ≃ 9% occupancy of the total Fe/Pt interfacial area, can be induced at the termination sites of threading dislocations of the Fe layer. The Fe/Pt interface significantly increases the coercivity and the saturation field, while the nanoparticles act as pinning centers for the magnetic domains’ rotation. At lower temperatures, the nanoparticles are actively coupled to the Fe/Pt system introducing an exchange bias effect. Spin pumping measurements below the blocking temperature of the nanoparticles reveal a suppression of spin rectification effects that is indicative of the important role of the Fe_3_O_4_ nanoparticles in spin current transmission through the interfaces.

The successful fabrication and the observed interaction of a small portion of magnetite particles embedded in the FM/NM interface, along with the surrounding spin system can define alternative ways to manipulate interfaces by introducing Fe-based ferri- and anti-ferromagnetic particles at the FM/NM interfaces. Such tunable interfaces may offer broad flexibility in future spintronic device design.

## Data Availability

The data presented in this study are available on request from the corresponding author.

## Supplementary Materials

The following are available online at https://www.mdpi.com/article/10.3390/ma14164354/s1, Figure S1: (a) HRTEM image of the multilayer along the [011]MgO/[001]Fe/[011]Pt zone axis. Two interfacial pits, denoted by arrows, present atomic structure. (b) The same HRTEM image, where the Fe layer spatial frequencies are digitally subtracted. The pits present either the Pt atomic structure (left) or no structure at all (right), and thus it is void. (c) GPA phase image of the multilayer, where only the original structures (MgO, Fe, Pt) are clearly visible. (d) GPA average strain map of the HRTEM image with the MgO structure as reference. No structure other than the original ones is detected. (e) The corresponding FFT diffractogram of the HRTEM image. The 111-type spatial frequencies of MgO (red) and Pt (blue) and the 110-type spatial frequency of Fe (green), used in GPA to produce the phase image and strain map, are shown in the dashed rectangle. (f) Line profile of the average strain shown in (d), depicting misfit strain values that are consistent with theoretical calculations of a relaxed multilayer configuration.
