# Supplementary material for: Magnetization Reversal and Dynamics in Epitaxial Fe/Pt Spintronic Bilayers Stimulated by Interfacial Fe3O4 Nanoparticles
Source: Materials (Basel). 2021 Aug 4;14(16):4354. doi: 10.3390/ma14164354 (PMC8401877; doi:10.3390/ma14164354)
Supplement: Supplementary file 1 [file materials-14-04354-s001.zip › materials-1281900-supplementary.pdf]

materials

MDPI

Supplementary Material

Magnetization Reversal and Dynamics in Epitaxial Fe/Pt Spintronic Bilayers Stimulated by Interfacial Fe3O4 Nanoparticles

Thomas Kehagias [1,]*, Dimitrios Karfaridis [1], Camillo Ballani [2], Laura Mihalceanu [3], Christoph Hauser [2], Isaak G. Vasileiadis [1], George P. Dimitrakopulos [1], George Vourlias [1] and Evangelos Th. Papaioannou [2]

[1] Physics Department, Aristotle University of Thessaloniki, 54124 Thessaloniki, Greece; dkarfari@physics.auth.gr (D.K.); isvasile@physics.auth.gr (I.G.V.); gdim@auth.gr (G.P.D.); gvourlia@auth.gr (G.V.)

[2] Institute of Physics, Martin-Luther University Halle-Wittenberg, 06120 Halle, Germany; camillo.ballani@physik.uni-halle.de (C.B.); Chris-Hauser90@web.de (C.H.); evangelos.papaioannou@physik.uni-halle.de (E.T.P.)

[3] Department of Physics, Technical University of Kaiserslautern, 67663 Kaiserslautern, Germany; mihalcea@rhrk.uni-kl.de

* Correspondence: kehagias@auth.gr

1. Detailed Experimental Methods

Samples were grown on MgO(100) substrates by electron-beam evaporation in ultra-high vacuum (UHV) conditions, namely under a base pressure of 4×10^{-10} Torr. Initially, the substrate's surface was chemically cleaned by ultrasonic bath in acetone and isopropanol for 5 min successively, followed by physical cleaning inside a high vacuum chamber, by annealing at 650°C for 1 hour, and by plasma-etching processing by a 50% mix of Ar-O_2 gas for 10 min. The bilayers were deposited at a substrate temperature of 450°C, followed by 30 min annealing at the corresponding growth temperature. In every case, the deposition rate of the electron gun consistently controlled at 0.3 nm/s, resulting in a Fe(12 nm)/Pt(10 nm) bilayer configuration.

Nanostructural properties of the interfacial structures were investigated by High-Resolution Transmission Electron Microscopy (HRTEM), in a Jeol 2011 UHR electron microscope, with a 0.19 nm point resolution and C_s=0.5 mm, operated at 200 kV. Specimens for cross-sectional HRTEM observations were prepared by the standard sandwich technique, followed by automated tripod polishing and final thinning to electron transparency by mild Argon ion (Ar^+) milling in the Gatan PIPS. Complementary TEM observations were carried out on specimens prepared in plan-view geometry to elucidate the distribution of nanoparticles at the Fe/Pt interfacial area.

X-ray Photoelectron Spectroscopy (XPS) analysis of the samples was performed on a Kratos Analytical AXIS Ultra^{DLD} system, with an Aluminum monochromatic X-Ray source (E_{photon} = 1486.7 eV), under 10^{-8} Torr of pressure inside the analysis chamber. The spectra were corrected in terms of charging, according to the C *-1s* peak at 284.6 ±0.2 eV of binding energy (B.E.), from the environmentally contaminated top-surface and of the sputtered-implanted Ar ions, during the etching process, at B.E.s of 242 ±0.2 eV for their $2p_{3/2}$ orbitals. The etching process was performed by an Ar^+ source applying a nominal etching rate of 100.12 Å/min on ion-gun (manufacturer), under 4 kV of accelerating voltage. The etching rate was corrected by calculations considering the different sputtering yields due to the mean atomic mass of each element (layer) [1,2]. Wide-scan spectra (full range) were recorded with passing energy of 160 eV, while High-Resolution (HR) regions with passing energy of 20 eV during a three-sweep scan. The fitting process was performed via the software Vision 2.2.10 developed by Kratos Analytical, using Shirley baseline to subtract the background from the photoelectron peaks and a combination of Gaussian/Lorentzian distributions, depending on the bonds. The magnetic anisotropy and static magnetic properties were probed by magneto-optical Kerr effect microscopy in the longitudinal alignment (L-MOKE). The wavelength of the polarized laser beam, used for the Kerr-angle detection, was 635 nm, while the sample was placed in the magnetic field of 200 mT, parallel to the incidence plane. The sample holder used was rotating around the vertical axis during the detection of the re-polarized reflected beam, varying the in-plane geometry of the samples, and magnetic hysteresis loops were recorded in the longitudinal geometry for all the crystallographic in-plane axis of the samples. Using the method, we extracted the coercive fields H_C as a function of the in-plane angle. The low-temperature magnetic characterization was done with a Quantum Design SQUID VSM magnetometer.

For the dynamic magnetic study, we performed inverse spin Hall effect (ISHE) measurements. Ferromagnetic resonance (FMR) was excited by microwave magnetic field h_{rf} induced by a coplanar waveguide. The Pt layer was facing the antenna using a thin insulating layer between. A tunable static external magnetic field H was applied in the x-y plane [(see Figure 8(a)]. The microwave antenna generates a dynamic magnetic Oersted field oriented perpendicular to the external field. Having electrical contacts along the y-direction, perpendicular to the externally applied magnetic field H and the antenna, the sample is connected to a lock-in amplifier for voltage detection. When the FMR condition is reached, a spin current gets injected at the FM/NM interface, from the FM into the NM, generating a charge current perpendicularly oriented to H via the ISHE. Using the lock-in amplifier, we recorded the generated V_{DC} voltage in the y-direction. To avoid parasitical voltages from the anisotropic magnetoresistance (AMR) and anomalous Hall effect (AHE), we present the ISHE voltage measurements for $\varphi = 0^{o}$, where the latter effects almost vanish, while the ISHE is at its maximum [3-5].

2. Phase and Strain Analysis by GPA

In order to determine the content of the Fe/Pt interfacial pits, we have employed Geometric Phase Analysis (GPA) on experimental HRTEM images [6]. Figure S1(a) is a HRTEM image of the Fe/Pt interface with two interfacial pits (black arrows) exhibiting atomic structure, along the [011]MgO/[001]Fe/[011]Pt projection direction. To identify this structure, the spatial frequencies corresponding to Fe were digitally subtracted from the common FFT diffractogram [Figure S1(e)] and the result is shown in Figure S1(b). As viewed in Figure S1(b), the left-hand pocket exhibits only the Pt atomic structure, whereas in the right-hand pocket no structure is visible. This clearly suggests that the atomic structure observed in Figures S1(a) and (b) is a consequence of Fe and Pt overlapping projected structures, which are in front and/or behind the pits, depending on the cross-sectional cut of the TEM sample. Therefore, we deduce that the pockets are either filled with Pt or they are void. Moreover, the GPA phase image of the multilayer [Figure S1(c)], corresponding to the HRTEM image, does not show the presence of a different structure amid the Fe and Pt layers. The same stands for the average GPA strain map of the multilayer [Figure S1(d)], produced by the 111 spatial frequencies of MgO and Pt, in conjunction with the 110 spatial frequency of Fe, shown in the dashed rectangle of the FFT diffractogram of Figure S1(e). Figure S1(f) is the line profile of the average lattice strain in Figure S1(d), along the [100] growth direction, with the MgO structure taken as reference. Our theoretical calculations of a fully relaxed multilayer configuration resulted in misfit strains of -15.2% for MgO/Fe and -6.6% for MgO/Pt, using the d-spacing values corresponding to the abovementioned spatial frequencies and considering the relative rotations between the relevant lattice planes. The calculations are shown below:

Angle between (111)MgO^(110)Fe = 10°

$$\varepsilon_{MgO/Fe} = \frac{\frac{d_{110}Fe}{cos10^{o}} - d_{111}MgO}{d_{111}MgO} = \frac{\frac{2.027\text{Å}}{0.9848} - 2.4266\text{Å}}{2.4266\text{Å}} = -0.152$$

Angle between (111)MgO^(111)Pt = 2°

$$\varepsilon_{MgO/Pt} = \frac{\frac{d_{111}Pt}{cos2^{o}} - d_{111}MgO}{d_{111}MgO} = \frac{\frac{2.265\text{Å}}{0.9994} - 2.4266\text{Å}}{2.4266\text{Å}} = -0.066$$

The average experimental strain values shown in the line profile of Figure S1(f) meet with the theoretical values and hence, an essentially stress-free configuration is anticipated. Moreover, the GPA phase image and strain map designate the stepped MgO/Fe interface.

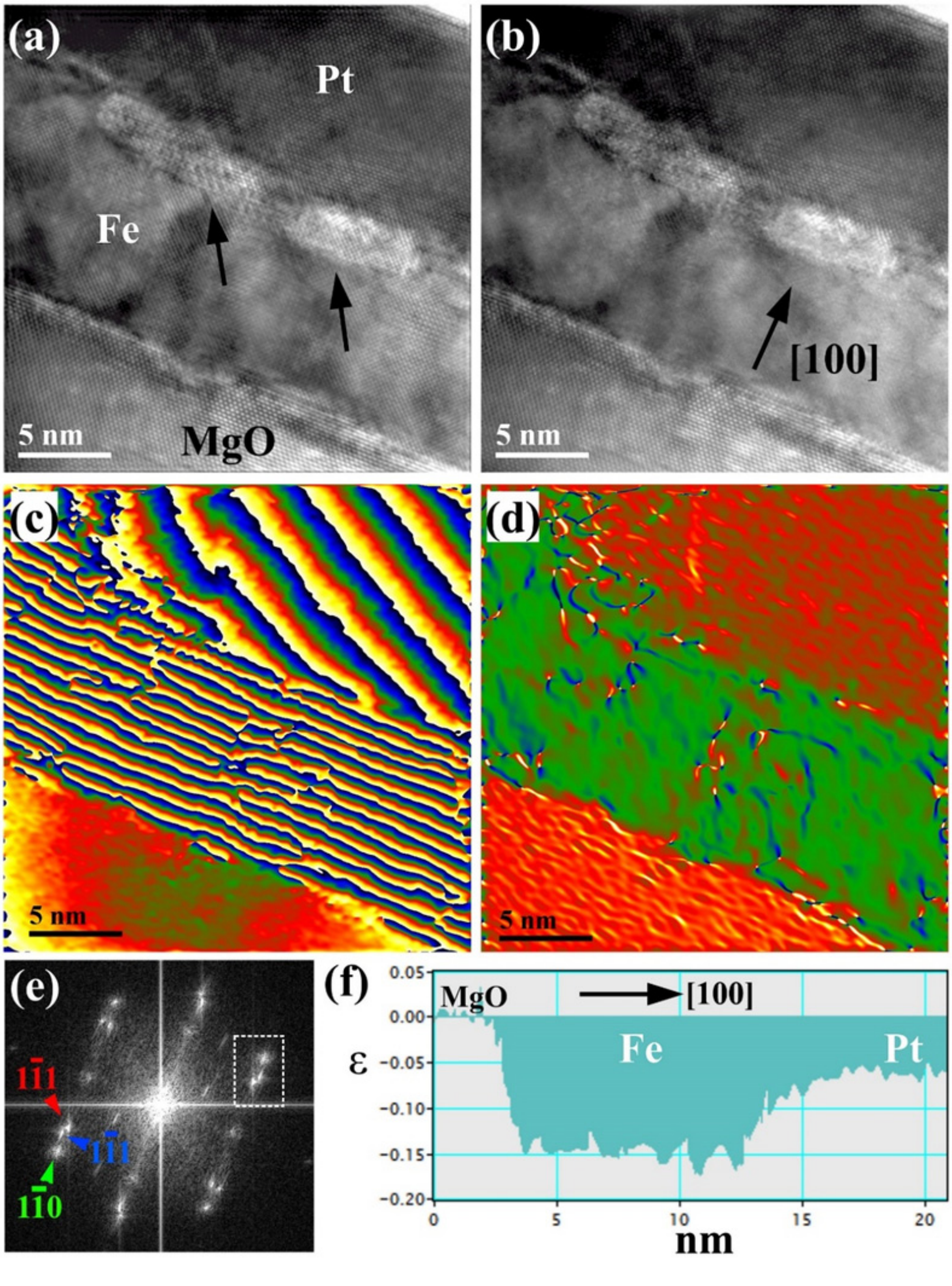

Figure S1. (a) HRTEM image of the multilayer along the [011]MgO/[001]Fe/[011]Pt zone axis. Two interfacial pits, denoted by arrows, present atomic structure. (b) The same HRTEM image, where the Fe layer spatial frequencies are digitally subtracted. The pits present either the Pt atomic structure (left) or no structure at all (right), and thus it is void. (c) GPA phase image of the multilayer, where only the original structures (MgO, Fe, Pt) are clearly visible. (d) GPA average strain map of the HRTEM image with the MgO structure as reference. No structure other than the original ones is detected. (e) The corresponding FFT diffractogram of the HRTEM image. The 111-type spatial frequencies of MgO (red) and Pt (blue) and the 110-type spatial frequency of Fe (green), used in GPA to produce the

phase image and strain map, are shown in the dashed rectangle. (f) Line profile of the average strain shown in (d), depicting misfit strain values that are consistent with theoretical calculations of a relaxed multilayer configuration.

References

[1] Cumpson, P.J.; Portoles, J.F.; Barlow, A.J.; Sano, N. Accurate argon cluster-ion sputter yields: Measured yields and effect of the sputter threshold in practical depth-profiling by x-ray photoelectron spectroscopy and secondary ion mass spectrometry. *J. Appl. Phys.* **2013**, *114*, 124313.

[2] Seah, M.P.; Nunney, T.S. Sputtering yields of compounds using argon ions. *J. Phys. D. Appl. Phys.* **2010**, *43*, 253001.

[3] Keller, S.; Greser, J.; Schweizer, M. R.; Conca, A.; Lauer, V.; Dubs, C.; Hillebrands, B.; Papaioannou, E. T. Relative Weight of the Inverse Spin-Hall and Spin-Rectification Effects for Metallic Polycrystalline Py/Pt, Epitaxial Fe/Pt, and Insulating YIG/Pt Bilayers: Angular Dependent Spin Pumping Measurements. *Phys. Rev. B* **2017**, *96*, 024437.

[4] Conca, A.; Heinz, B.; Schweizer, M. R.; Keller, S.; Papaioannou, E. T.; Hillebrands, B. Lack of Correlation between the Spin-Mixing Conductance and the Inverse Spin Hall Effect Generated Voltages in CoFeB/Pt and CoFeB/Ta Bilayers. *Phys. Rev. B* **2017**, *95*, 174426

[5] Harder, M.; Gui, Y.; Hu, C.-M. Electrical Detection of Magnetization Dynamics via Spin Rectification Effects. *Phys. Rep.* **2016**, *661*, 1–59.

[6] Hÿtch, M.; Snoeck, E.; and Kilaas, R. Quantitative Measurement of Displacement and Strain Fields from HREM Micrographs. *Ultramicroscopy* **1998**, *74*, 131–146.
